# Optimizing regional rare disease management systems within China’s policy framework: practical insights from the shantou experience

**DOI:** 10.1186/s13023-026-04340-3

**Published:** 2026-04-01

**Authors:** Beiyan Wu, Yali Wang, Chao Liu, Qilin Wang, Mengchun Gong, Yongsong Chen

**Affiliations:** 1 The First Affiliated Hospital of Shantou University Medical College, Guangdong, China; 2 DHC Technologies, Beijing, China; 3 Guangdong Medical University, Guangdong, China

**Keywords:** Shantou experience, Rare disease drug supervision, Rare disease treatment, Medical insurance access

## Abstract

**Background:**

Rare diseases refer to those that have an extremely low incidence within a specific region or population and are frequently hereditary, chronic, severe, and disabling. Globally, approximately 7,000 distinct rare diseases exist, affecting the health and quality of life of over 300 million individuals. In China, patients with rare diseases are in an especially critical predicament, confronted with multiple challenges such as difficult diagnoses, unclear treatment options, high costs of drug treatment, and insufficient social support systems. The presence of these issues not only exacerbates the physical and mental burden of patients but also has a profound influence on their families and society. Therefore, enhancing the diagnosis and treatment level of rare diseases, improving the social security system, and reducing the economic pressure on patients have become urgent matters in the current medical and health domain in China.

**Result:**

Through an in-depth analysis of relevant policies and research in China, we reach the conclusion that the four key challenges encountered in the treatment of rare diseases in China are: Incomplete social support system, Imperfect healthcare services, Incomplete medical insurance and Inadequate access to medicines. Next, this paper takes the “Shantou Experience” extracted from the practice of rare disease diagnosis and treatment by the First Affiliated Hospital of Shantou University Medical College as a case to discuss the innovative measures adopted in optimizing the management mode and treatment methods of rare disease drugs. It also summarizes three major problems that urgently need to be solved in the treatment of rare diseases in China, with the aim of providing a reference for further enhancing the medical security system of rare patients.

**Conclusion:**

This paper comprehensively summarizes four major challenges confronted by China in the domain of rare disease diagnosis and treatment, and elaborates on the practice of “Shantou Experience” in enhancing the accessibility of rare disease drugs and innovating rare disease medical models. Meanwhile, the paper also proposes three perspectives on the future development direction of rare disease treatment in China.

## Background

Rare diseases are generally characterized by their low prevalence and the limited number of individuals affected within a specific region or population [1]. According to data from the World Health Organization, there are an estimated 5,000 to 8,000 known rare diseases across the globe, which account for roughly one-tenth of all human diseases and affect over 300 million lives [2]. These conditions are often chronic, progressive, and debilitating, leading to significant morbidity and mortality [2, 3]. The heterogeneous, complex, and individually rare nature of rare diseases makes them difficult to diagnose and challenging to assess collectively. However, with the continuous progress in biological sciences, an increasing number of orphan drugs are being developed, offering hope and improving the condition and quality of life for numerous patients with rare diseases.

As one of the most populous countries worldwide, China is also among those with a large number of rare disease patients. According to statistics, there are approximately 25 million rare disease patients in China, constituting 1.8% of the total population [4]. However, China’s attention and support for rare diseases started relatively late and is still in the fledgling stage. In China, there are no specific laws and regulations for rare diseases, and less than 200 rare diseases have clear diagnostic criteria and treatment plans [5, 6]. Moreover, the majority of specialized drugs for rare diseases rely on imports, which are expensive and not easily covered by medical insurance. In recent years, the National Health Commission has actively promoted the linkage of medical care, medical insurance and medicine, continuously improved relevant policies, enhanced the diagnosis and treatment capabilities of rare diseases, and ensured the supply of drugs. Although the government has increased its support for rare diseases, it still needs to further strengthen policy support in terms of drug approval, access system and drug popularization, accelerate the process of drug marketing, and ensure that drugs can reach and benefit patients. Generally, patients with rare diseases in China still face challenges in diagnosis, treatment, economic pressure, insufficient social support and other issues, and there is an urgent need to intensify policy guidance and social care.

This paper will initially outline the current challenges encountered in the diagnosis and treatment of rare diseases, and then take the “Shantou Experience” as a case to elaborate on its innovative approaches in the drug management model and disease diagnosis model in the diagnosis and treatment of rare diseases, and ultimately anticipate the future development trend of China’s rare disease treatment.

## Main text

### Challenges in treatment of rare diseases in China

**Fig. 1:**
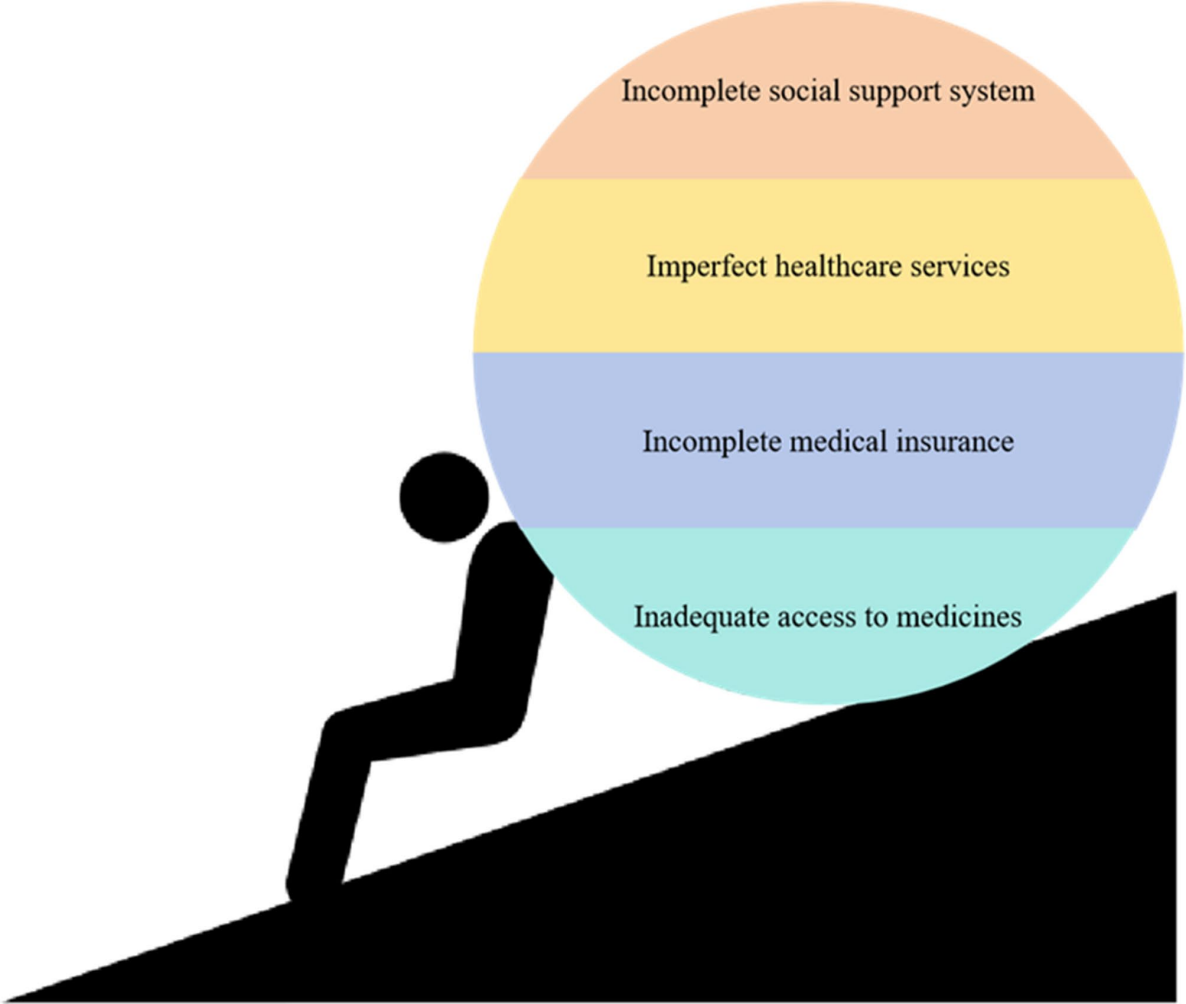
Four challenges faced by rare disease patients in China

Although the number of individual patients with rare diseases is not large, as a group, rare disease patients share common characteristics and face similar challenges [7]. Globally, the medical needs and social support for patients with rare diseases and their families are generally insufficient [8]. According to statistics, approximately half of suspected rare disease patients remain undiagnosed, and those who are diagnosed often face delayed diagnosis, inappropriate treatment, inadequate care, and low social acceptance [8]. In an era of limited resources and tight budgets, evidence regarding the economics of health plays a crucial role in guiding policymakers in the rational allocation of resources [9–11]. Compared to common diseases, the number of patients with rare disorders is small, and they are often overlooked by the government, health institutions, pharmaceutical companies, and even the public [12]. This directly results in the basic needs of patients with rare diseases in medical treatment and daily life failing to receive due attention and protection. On the other hand, due to the late initiation of rare disease research in China, coupled with unique systemic and structural characteristics, China’s rare disease care faces distinct dilemmas beyond global common challenges: the absence of specialized laws and regulations for rare diseases, pronounced regional disparities in medical resources coupled with weak grassroots diagnostic capabilities, unmet reimbursement needs for ultra-expensive biologics within the medical security system, and heavy reliance on imported drugs alongside insufficient incentives for domestic orphan drug development. These specific predicaments further exacerbate the difficulty of diagnosis and treatment for Chinese rare disease patients, with several key issues in China’s rare disease care detailed as follows (Fig. 1):

#### Incomplete social support system

Social support system pertains to those social elements that are intimately associated with the physical well-being of individuals, and it embodies the conduct and attitude of society in offering assistance to individuals [13–15]. For rare disease patients, the social support system encompasses a variety of services. These include, but are not limited to, societal care for those living with rare conditions, legislation to protect the rights and interests of patients with rare diseases, vocational assistance to enhance employment opportunities, and psychological counseling to address the emotional and mental health needs of the patients [16–18]. Nevertheless, the present social support system for patients with rare diseases in China is profoundly insufficient to meet the actual requirements of this cohort. Specifically, China has not yet promulgated laws and regulations specifically targeting rare diseases, thereby giving rise to the absence of a solid legal foundation for safeguarding the rights and interests of patients with rare diseases [6]. Concerning medical resources, the limited quantity of specialized hospitals and their uneven geographical distribution render it arduous for numerous patients with rare diseases to obtain timely and efficacious medical services [19]. Additionally, the society’s awareness of rare diseases is generally low, and the public’s comprehension of rare diseases is inadequate, resulting in a low degree of social attention and support [20]. The deficiency of the social support system not only exposes rare patients to the challenge of the disease but also burdens them with pressure from all societal levels, which undoubtedly exacerbates the predicament of rare patients [20].

#### Imperfect healthcare services

In China’s vast territory, pronounced disparities in economic development and medical resource distribution persist across regions, despite national efforts to optimize the system. Since the 13th Five-Year Plan period, China has established a national rare disease diagnosis and treatment collaboration network covering 324 hospitals (adopting a three-tier structure: 1 national leading hospital, 32 provincial leading hospitals, and 291 member hospitals) and issued the Rare Disease Diagnosis and Treatment Guidelines (2019 Edition) to standardize clinical practices. However, these initiatives have not fully addressed accessibility gaps at the grassroots level. Such regional imbalances lead to insufficient awareness and understanding of rare diseases among both the public and healthcare professionals—particularly in primary care settings—directly compromising the accuracy and timeliness of clinical diagnoses [21, 22]. Consequently, prolonged diagnostic timelines, delayed treatments, and misdiagnoses remain prevalent. A national survey of 258 medical staff at top-tier (tertiary) hospitals revealed that 33.3% admitted to having no knowledge of rare diseases, while 1.1% had never heard of them [23]. Furthermore, another relevant survey covered 27 out of China’s 34 provincial administrative regions, thus ensuring broad geographic representativeness. Among the 539 surveyed emergency physicians, over 74.58% (402/539) of respondents were from tertiary hospitals (including Tertiary A and B hospitals), and nearly half (45.27%) had more than 15 years of clinical experience. Among these respondents, 98.5% rated their knowledge of rare diseases as “minimal” or “insufficient,” with only 0.19% considering themselves “well-informed” and 1.30% “moderately knowledgeable“ [24, 26]. Obviously, compared with senior physicians in tertiary hospitals, the situation among junior staff in primary healthcare facilities is even more pronounced—primarily due to their limited access to standardized guidelines and technical support. Investment in rare disease research in China is inadequate when compared to more common conditions [19, 25, 26]. There is a scarcity of specialized laboratories and researchers, as well as a limited comprehension of the pathogenesis of rare diseases [20]. These factors are substantial barriers to the advancement of rare disease drug research and development within the country.

#### Incomplete medical insurance

Research indicates that the medical security system for rare diseases is highly correlated with the social economy [9]. While China’s per capita income level remains below that of developed countries, significant multi-departmental coordination efforts have emerged in recent years—including NMPA’s accelerated approval pathways (accepting single-arm trials and bridging studies for conditional approval) and joint tax incentives by the Ministry of Finance and NMPA (54 formulations exempted from VAT as of 2022) [27, 31], alongside NHSA’s higher willingness-to-pay thresholds in access negotiations. Despite these advances, systemic challenges persist in balancing cost control with innovation incentives across departments. On the other hand, the cost-sharing mechanism for rare diseases is incomplete. One of the core problems is that the basic medical insurance system has not fully exerted its function, resulting in some rare disease patients not being covered. China’s medical security system is based on medical insurance, supplemented by commercial insurance and charitable funds. However, even if rare disease drugs are included in the National Medical Insurance Catalog, they can only be partially guaranteed by the state. Analysis reveals that approximately 67% of rare disease drugs marketed in recent years (50 of 75 drugs as of June 2023) have been included in the National Medical Insurance Catalog [27, 28, 30], yet this progress has primarily benefited lower-cost therapies, while ultra-expensive biologics (> 1 million RMB annually) remain excluded. The complementary role of commercial insurance and social charitable organizations in the medical security of rare diseases has not been fully manifested. Collectively, these issues have led to a medical security gap for patients with rare diseases, augmenting their economic burden and influencing their quality of life.

#### Inadequate access to medicines

China’s rare disease drug market is mainly dependent on imported originator drugs and domestic generic drugs [29], and policies also encourage domestic enterprises to independently develop originator drugs. The exorbitant cost of imported originator drugs, combined with the restricted coverage of rare disease treatment in China, has resulted in low reimbursement rates for treatment expenses, rendering it arduous for numerous patients with rare diseases to afford. As of 2020, five rare disease drugs with confirmed annual treatment costs in China exceeded 1 million RMB per patient (e.g., elosulfase alfa for mucopolysaccharidosis IVA at 2.92 million RMB and agalsidase beta for Fabry disease at 1.39 million RMB), with seven more projected to do so upon market entry [30]. After charitable assistance, costs are comparable to overseas levels (USD 200,000–400,000), confirming a global challenge. However, while these therapies are fully reimbursed in countries like Russia, Australia, and Japan, none were covered by China’s National Reimbursement Drug List as of 2020, forcing most registered patients to forego treatment. The advancement of domestic generic drugs is relatively straightforward: they do not require clinical trials and only need to complete bioequivalence verification. However, an analysis of domestic generic drugs shows their limited impact: as of 2020, they covered less than 5% of rare disease indications, mainly older small-molecule drugs, and no biosimilars were available for high-priced biologics (such as enzyme replacement therapies) that constitute the core economic burden [30].Although the state encourages domestic enterprises to develop rare disease drugs through initiatives such as priority review and approval [31, 32], market exclusivity periods, and special funds for rare disease R&D, the high R&D costs (100 million to 200 million RMB) and extremely small patient populations (fewer than 500 patients per disease) result in negative return on investment (ROI), leaving domestic manufacturers with insufficient motivation for R&D.The small patient population complicates the recruitment for clinical trials, which constraints research timelines and extends development cycles. Although China has streamlined the approval process for rare disease drugs, stringent regulatory procedures still entail considerable time. This aggravates the difficulty of R&D and promotion of domestic originator drugs in China. Under China’s healthcare system, where institutions provide both medical services and sell drugs, doctors may be inclined to favor options that are more advantageous, thus distorting the authenticity of the drug market and potentially leading to adverse selection or moral hazard. Information asymmetry may also encourage pharmaceutical companies to focus on the more stable markets for common drugs, neglecting the research and development of novel and orphan drugs, ultimately resulting in a market imbalance and failure in resource allocation.

Notably, nusinersen became a pioneering case for ultra-high-cost rare disease drugs accessing national medical insurance: in 2021, it was included in the NRDL through national medical insurance negotiations, with its annual treatment cost drastically reduced from 1.05 million RMB to approximately 33,000 RMB. This breakthrough laid a policy foundation for the subsequent dynamic adjustment of high-value orphan drugs into medical insurance. By 2026, alglucosidase alfa is covered by national unified medical insurance negotiations, while imiglucerase and agalsidase beta adopt the model of “national separate payment + local limited reimbursement” in select regions [33]. Notably, it is commendable that these phased progresses have not only brought life-saving therapies within reach for tens of thousands of patients but also demonstrated China’s proactive efforts to refine the multi-level protection system for rare diseases—reflecting a pragmatic balance between medical insurance affordability and patient needs. However, constrained by multiple factors such as the sustainability of medical insurance funds, the high R&D costs of orphan drugs, and uneven regional implementation capacities, the national medical insurance system still cannot include all high-value rare disease drugs in the catalog.

In the process of advancing from “no national protection” to “stratified and targeted protection”, China’s rare disease work continues to face numerous unresolved challenges. Domestic generics cover only a small share of rare disease indications, while domestic innovative drugs are plagued by development dilemmas. Coupled with partial progress and lingering hurdles in updated policy frameworks, these issues underscore the arduousness of refining China’s rare disease protection system, calling for sustained and targeted efforts to break through existing bottlenecks.

### Shantou experience - a new model of drug management for treatment of rare diseases in China

**Fig. 2:**
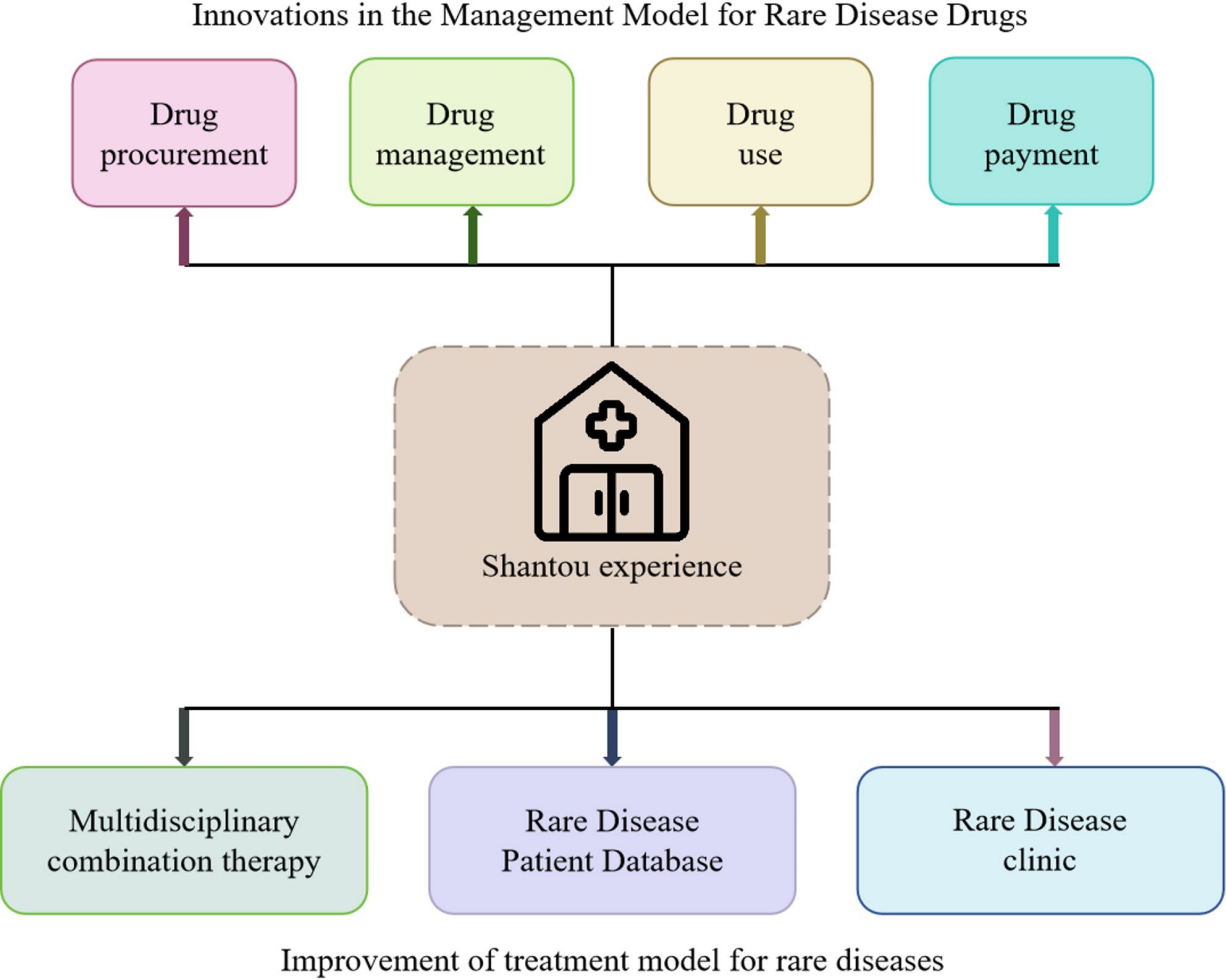
Shantou experience -- the improvement strategy of drug management model and treatment model for rare diseases

Since 2016, the medical industry in China has achieved remarkable advancements in the domain of prevention and treatment of rare diseases. A multitude of hospitals have intensified their endeavors to advance epidemiological research on rare diseases, facilitate in-depth diagnosis, prevention and treatment, and actively explore and develop state-of-the-art diagnosis and treatment technologies associated with rare diseases. Among them, the First Affiliated Hospital of Shantou University Medical College has made outstanding contributions through the “Shantou Experience” summarized from the practice of rare disease diagnosis and treatment, and has received high praise from the Chinese government. This article will take Shantou Experience as an example to conduct in-depth analysis of its innovative measures and major improvement strategies in the treatment of rare diseases and drug management (Fig. 2).

#### Innovation of drug management model for rare diseases

The First Affiliated Hospital of Shantou University has implemented medical system innovation in the four key aspects of procurement, management, use and payment of rare disease drugs, significantly enhancing the accessibility and affordability of rare disease drugs in China.

In the procurement of rare disease drugs, the Shantou Experience adopts the clinical-driven rapid application process to simplify the drug approval steps. Once the clinician puts forward the demand, the procurement process commences directly, forming a closed-loop drug supply system encompassing procurement, acceptance, warehousing, storage, secondary pharmacy, pharmacist dispensing to clinical use. Compared with other drug approval models, this process in the Shantou Experience has eliminated the protracted approval and bidding links, enabling a closer integration of drug supply with the front-line clinical demands and effectively preventing the issue of mismatch between drugs and the actual needs of patients. This model not only enhances the pertinence and efficiency of drug supply but also offers a more substantial guarantee for the treatment of patients with rare diseases. This model has established a rare disease drug library with the most comprehensive variety, the largest reserves, the strongest supply and the most professional service in eastern Guangdong, achieving full coverage of rare disease drugs by medical insurance and expanding the service scope to 13 million resident population, covering the three cities in eastern Guangdong.

In terms of drug management, the Shantou Experience has innovatively established a “rare disease access model”, creating a comprehensive supervision system for rare disease drugs, covering the entire process of diagnosis, decision-making, utilization and post-use evaluation, and providing precise medical services for patients. The hospital’s intelligent pharmacy management system and pharmacists’ prescription review intervention system update drug information regularly to ensure drug safety. Additionally, the hospital has set up multiple rare disease diagnosis and treatment centers in eastern Guangdong, improving the efficiency of diagnosis and treatment and facilitating patients’ medical treatment.

In terms of drug utilization, the hospital promoted the close collaboration between pharmacists and the clinic, and improved the quantity and quality of pharmaceutical consultations. For example, for idiopathic pulmonary hypertension, a comprehensive drug evaluation was conducted to guide rational clinical drug use and promote the development of clinical work. The hospital also implemented a doctor-patient joint drug supervision model, and pre-approved prescriptions through the pharmaceutical information system to ensure drug safety. After treatment, patients and family members participate in drug monitoring, providing support for home care and recovery for long-term treatment through active reporting mechanisms.

In terms of drug payment, the Affiliated Hospital of Shantou University has innovated local implementation strategies within the national “dual-channel” policy framework (established by China’s National Healthcare Security Administration in 2021), which explicitly identifies rare disease drugs as a key priority for “dual-channel” coverage to address the gap between national policy approval and local patient access [34]. The Shantou Experience’s contribution lies in innovating its local implementation through two key measures: (1)through the panel discussion, prompting the government medical insurance department to establish special payments for rare diseases and promote multi-channel payments such as commercial medical insurance, public welfare funds and charity funds, initiating SMA treatment; (2)establishing a hospital-pharmacy-payer coordination platform that reduced drug dispensing time; (3)facilitating cross-regional insurance claims that facilitated reimbursement for 60% of SMA patients who were from outside the Shantou area. While the “dual-channel” policy enables nationally-negotiated drugs to be reimbursed through both hospital and retail pharmacy channels, the Shantou model demonstrates how hospitals can operationalize this policy through integrated payers-physician-pharmacist collaboration, charitable foundation coordination, and digital prescription tracking systems. This implementation optimization has increased the actual insurance payment ratio for rare disease drugs from 55% to 78% in our service region, offering a transferable model for reducing the implementation lag between national policy and patient benefit.

#### Improvement strategy of treatment mode for rare diseases

Besides the enhancement of the management model for rare disease drugs, the innovation in the treatment model of rare diseases has significantly alleviated the pressure on patients with rare diseases. In the realm of innovating the treatment mode for rare diseases, the Shantou Experience has notably alleviated the medical burden of patients with rare diseases. Its core highlights are manifested in the establishment of a multidisciplinary joint diagnosis and treatment model and a patient-centered diagnosis and treatment model. Relying on its high-level multidisciplinary team, the hospital has constructed a database of rare disease experts encompassing specialties such as internal medicine, surgery, pediatrics, neurology, prenatal diagnosis, and others, and has established a specialized rare disease diagnosis and treatment center. Through regular multidisciplinary discussions, these centers customize the optimal diagnosis and treatment for complex cases.

The rare disease diagnosis and treatment center fully utilizes precision medicine technologies, such as genetic counseling, prenatal genetic testing, gene sequencing, and bioinformatics analysis, to achieve accurate diagnoses of patients and provide professional genetic counseling and fertility guidance for high-risk groups to facilitate the tertiary prevention of rare diseases. In 2022, the establishment of the hospital’s prenatal diagnostic center enabled high-risk fetuses of couples with homothalassemia carriers to be screened through amniocentesis genetic testing, effectively identifying severe thalassemia fetuses and significantly reducing the incidence of hereditary rare diseases.

Based on the attention paid to the needs of patients, the hospital has created a database and archives for rare patients and, through regular follow-up guidance, can promptly grasp the changes and requirements of patients. In collaboration with the Association of rare disease patients and volunteer groups, the hospital has conducted publicity, education, psychological counseling, and mutual assistance activities to enhance the quality of life of patients. In 2022, with the reduction in the price of drugs for treating Fabry disease and their inclusion in the national medical insurance, the neurology department of the hospital successfully procured drugs for patients and carried out enzyme replacement therapy, significantly improving the condition and quality of life of patients.

Furthermore, the hospital has established a special disease channel for rare patients, simplifying the treatment process, reducing waiting time, and further enhancing the medical experience. The innovative measures in the treatment mode of rare diseases in the First Hospital of Shantou not only enhance the efficiency of diagnosis and treatment but also offer patients more humanized and precise medical services.

#### Generalizability of the shantou experience and its adaptation path at the national level

As a prefecture-level regional medical center, rather than a provincial core medical carrier, the First Affiliated Hospital of SUMC has formed an innovative model with unique promotion value. Its practices are highly consistent with China’ national rare disease prevention and treatment framework, effectively addressing the systemic challenges faced by rare disease management in China, and building a feasible path for scaling up local experiences nationwide.

Playing a pivotal connecting role, this model bridges provincial high-quality medical resources and national policy guidelines with grassroots and remote areas, perfectly adapting to China’s reality—numerous grassroots hospitals with weak diagnostic capabilities and inconvenient medical access for patients in remote regions. Its generalizable core lies in three aspects. Firstly, under the national “dual-channel” policy, it optimizes drug procurement and medical insurance settlement, establishing a “green channel” for rapid procurement and unified reimbursement standards, which solves the “last mile” issue of policy implementation and is replicable across regions.

Secondly, the integrated “clinical-genetic-imaging-laboratory” diagnosis model and online MDT mechanism specifically tackle the uneven regional medical capabilities, providing practical solutions for remote areas radiated by prefecture-level centers [35]. Thirdly, the regional medical collaboration network led by the hospital, covering 13 million residents in eastern Guangdong, aligns with the construction goal of China’s national rare disease diagnosis and treatment network, verifying the value of prefecture-level centers in linking grassroots institutions.

The vitality of this experience comes from its in-depth adaptation to the national collaborative network [36]: it accurately implements top-level designs such as the two national rare disease catalogs (covering 207 diseases) and NMPA’s accelerated drug approval, and optimizes the three-level diagnosis and treatment network as a prefecture-level node. For promotion, we should adhere to the “stratified adaptation” principle [37]: provincial centers replicate policy optimization and MDT models; prefecture-level and grassroots institutions focus on screening and teleconsultation. As the national leading coordinating hospital in China’s rare disease field, Peking Union Medical College Hospital (PUMCH) is well-positioned to take the lead in integrating local experiences such as the Shantou Experience into the advancement of the national collaboration network and promoting of a the multi-level medical security model, thereby facilitating the organic connection between national centers, provincial centers and local centers. This will realize the effective integration of local practices with the national network coordinated by PUMCH, laying a solid foundation for subsequent development.

### Prospects for treatment of rare diseases in China

**Fig. 3:**
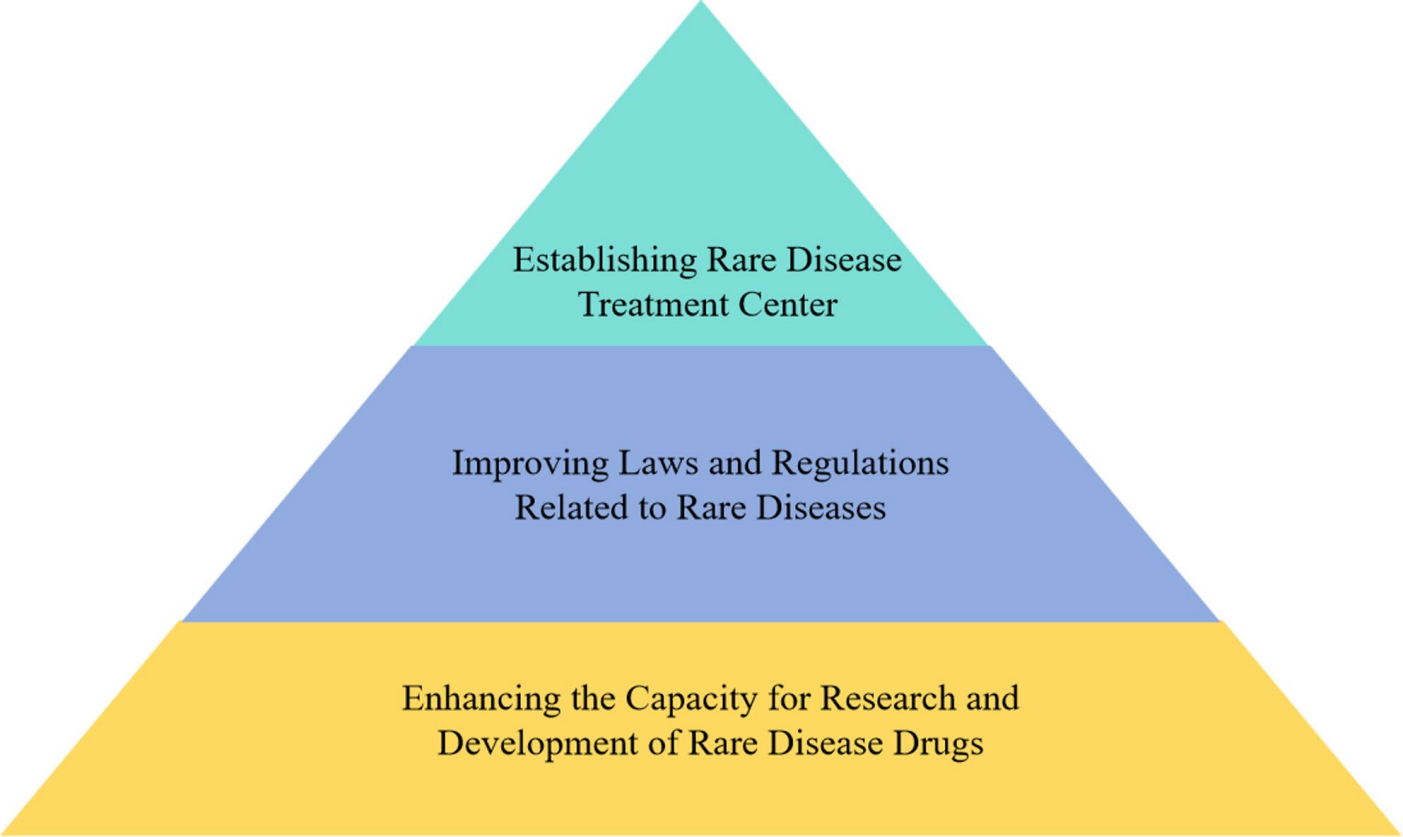
Important problems in rare disease diagnosis and treatment in China

China is increasingly taking the lead in clinical and scientific research on rare diseases globally. However, how to extensively benefit patients in remote and grassroots regions remains a challenge. This calls for Chinese hospitals to deepen the rare disease diagnosis and treatment system at the grassroots level and achieve close cooperation in multiple disciplines and aspects. The Shantou Experience has played a significant role in promoting the diagnosis and treatment of rare diseases in China. Through innovative diagnostic technologies, treatments, and drug management strategies, the Shantou Experience has successfully extended comprehensive and specialized healthcare services to patients with rare diseases in underserved areas. Looking ahead to the future of rare disease treatment in China, combined with the experience of the Shantou Experience and China’s specific social environment, the following aspects deserve further attention and efforts from medical workers (Fig. 3):

#### Establishing rare disease treatment center

Nestled in the eastern part of Guangdong Province, China, Shantou faces the hurdles of a somewhat secluded location, a less advanced economic landscape, and an infrastructure in need of enhancement. Yet, despite these challenges, Shantou Experience has provided valuable insights and recommendations for the prevention and management of rare diseases across China. Analyzing the achievements of Shantou Experience, we find that a dedicated team of rare disease experts and professional pharmacists has not only supported numerous districts and counties in Shantou and various hospitals but has also been instrumental in the fight against rare diseases. To bridge the gap in medical security capabilities across regions and to improve the diagnostic rate for rare diseases, it is imperative to establish local rare disease diagnosis and treatment centers within the existing collaborative network. These centers will serve as a unified platform for medical resources, fostering interregional research collaboration, establishing an online communication network, and effectively pooling expert knowledge and resources. Leveraging the rare disease departments of major hospitals, an information sharing platform can significantly streamline patient appointments, medication consultations, and treatment procedures. The local rare disease centers will maximize the benefits of resource integration, elevate the standard of care in the region, and lay a robust foundation for the advancement of prevention and treatment technologies, as well as in-depth academic research. Furthermore, these centers will encourage interdisciplinary teamwork, disseminate rare disease knowledge, and enhance the diagnostic and treatment capabilities of grassroots medical staff. Through these comprehensive strategies, we aim not only to deliver more accessible and efficient healthcare to patients with rare diseases but also to propel the overall development of rare disease prevention and treatment in China. In doing so, we will work towards a more equitable distribution of medical resources and a substantial upgrade in the quality of healthcare services.

#### Improving laws and regulations related to rare diseases

The medical security of patients with rare diseases encompasses numerous domains such as medical treatment, medical insurance, and medicine. While China has established an effective de facto definition mechanism through the First and Second National Rare Disease Catalogs, covering 207 diseases, the absence of codified legislation creates uncertainty for stakeholders. The catalog selection process—involving 24 expert panels reviewing epidemiology, disease burden, diagnosability, and treatability—provides a rigorous, evidence-based framework that has functioned as a practical standard for drug approval and reimbursement decisions. However, the policy-based nature of the current system presents implementation challenges: there is no clear legal definition of rare diseases or orphan drugs at the national level, and relevant provisions are scattered across various policies and regulations rather than being systematically integrated. The legal definition of orphan drugs serves as the basis for identifying rare diseases and is also the key to motivating pharmaceutical companies to invest in the research and development of orphan drugs. More importantly, with over 7,000 known rare diseases worldwide and approximately 1,400 in China, the 207 diseases in the catalog cannot cover all patient needs, leaving those with non-catalog rare diseases facing inadequate medical security. It is proposed that China draw upon the experience of international orphan drug legislation, commence from multiple aspects such as epidemiology, disease severity, and economy, and take into account China’s national conditions including the level of political and economic development, population size, and medical security capacity. The definition of rare diseases should be clarified at the legal level as soon as possible to lay a solid foundation for subsequent orphan drug legislation and ensure that the treatment of rare patients is governed by laws. China’s regulations concerning rare diseases mainly exist in the form of policies, and this flexible policy system may undermine the enthusiasm of research and development institutions and pharmaceutical enterprises. Laws are more stable than policies and are less prone to frequent changes. Therefore, only through a well-established legislative system to solidify the incentive policy for rare diseases can we enhance the trust of R&D institutions and pharmaceutical companies and protect their interests, thereby promoting the research and development and production of drugs for rare diseases. China’s legislation system related to rare diseases is not yet complete, and the absence of legislation often leads to the deficiency of lower-level laws, which affects the motivation of research and development institutions and pharmaceutical companies. Shantou Experience has accumulated a considerable amount of valuable experience in the practice of diagnosing and treating rare diseases, and these cases will provide significant empirical support for the formulation of laws and regulations related to rare diseases.

#### Enhancing the capacity for research and development of rare disease drugs

The research and production of orphan drugs constitute crucial elements of the medical security system for rare diseases. In the domain of rare disease drug research, a considerable number of studies remain in the preclinical stage or have not yet reached clinical trials, and the approved orphan drugs can only fulfill the treatment requirements of a small proportion of rare diseases [38]. Consequently, as high as 95% of rare disease patients encounter the predicament of having no accessible treatment. It is also notable that over one-third of the approved orphan drugs concentrate on oncology. In the future, transforming the basic research outcomes in rare diseases into clinical therapies should become a key focus area for public and charitable fund support. Hence, there is an urgent demand for more research and development funds and closer collaboration among various government agencies to stimulate the development of orphan drugs through policy measures. The Shantou Experience, via a multi-organization cooperation model, has efficiently connected the data of rare disease patients with pharmaceutical companies, achieving precise treatment in the therapeutic process of multiple patients. This modernized diagnosis and treatment plan has offered valuable experience for the treatment of rare diseases. Currently, major developed countries and regions worldwide have established rare disease patient registration systems to support clinical research and new drug clinical trials for rare diseases, such as genetic disease and rare disease information centers, the Orphanet rare disease and orphan drug website in the European Union, the National Organization for Rare Disorders in the United States, and the rare disease registration system in China. Looking ahead, how to establish and select scientific research approaches and how to fully explore and utilize existing real-world data will play a vital role in promoting the security of medication for rare diseases. Through these endeavors, China can provide more effective treatment options for rare disease patients, thereby enhancing their quality of life and life expectancy.

## Conclusion

With the advancement of science and technology, the enhancement of medical technology, the evolution of the social construction concept, and the strengthening of medical security, the supply of orphan drugs and the demands for diagnosis and treatment for patients with rare diseases and their families have witnessed significant improvement. Our government is actively seeking to establish a medical security system for rare diseases. Faced with the issues of low accessibility to orphan drugs, a weak research and development foundation of pharmaceutical enterprises, regional security disparities, and an incomplete medical security system, China is required to accelerate the legislation on medical security for rare diseases, enhance the incentive mechanism for pharmaceutical companies, establish local diagnosis and treatment centers, and construct a security system based on medical insurance, supplemented by commercial insurance and social cooperation. China’s clinical research on rare diseases is at the global forefront; however, it needs to delve deeper into grassroots levels and engage in multi-party collaboration to benefit more patients in remote areas. As a national rare disease cooperative network hospital, the First Hospital of Shantou University is dedicated to establishing a quality control center, reinforcing pharmaceutical management, offering comprehensive medical services, and addressing the challenges of poor information and accessibility. To enhance drug accessibility and affordability, the hospital will promote research, seek policy support, conduct thematic activities, create special funds, and call for social attention. Through deepening research, strengthening cooperation, and promoting policy reform, the First Hospital of Shantou University will provide better treatment opportunities and quality of life for patients with rare diseases, and its model is anticipated to offer a reference for the treatment efforts across the country.

## Data Availability

The data sources for this study encompass published reports and academic journal articles, and there are no limitations on data access or utilization.
